# Twenty-Four-Hour Urinary Sugars Biomarker in a Vending Machine Intake Paradigm in a Diverse Population

**DOI:** 10.3390/nu16050610

**Published:** 2024-02-23

**Authors:** Mary M. Ahern, Emma J. Stinson, Susanne B. Votruba, Jonathan Krakoff, Natasha Tasevska

**Affiliations:** 1 Obesity and Diabetes Clinical Research Section, Phoenix Epidemiology and Clinical Research Branch, National Institute of Diabetes and Digestive and Kidney Diseases, National Institutes of Health, Phoenix, AZ 85016, USA; maryahern01@arizona.edu (M.M.A.); emma.stinson@nih.gov (E.J.S.); votrubas@niddk.nih.gov (S.B.V.); jkrakoff@nih.gov (J.K.); 2 College of Health Solutions, Arizona State University, Phoenix, AZ 85004, USA

**Keywords:** sucrose, fructose, biomarker, epidemiology, urine, sugars intake, vending machine

## Abstract

Accurately measuring dietary sugars intake in large-scale epidemiological studies is necessary to understand dietary sugars’ true impact on health. Researchers have developed a biomarker that can be used to assess total sugars intake. Our objective is to test this biomarker in diverse populations using an ad libitum intake protocol. Healthy adult participants (*n* = 63; 58% Indigenous Americans/Alaska Natives; 60% male; BMI (mean ± SD) = 30.6 ± 7.6 kg.m^2^) were admitted for a 10-day inpatient stay. On day 2, body composition was measured by DXA, and over the last 3 days, ad libitum dietary intake was measured using a validated vending machine paradigm. Over the same days, participants collected daily 24 h urine used to measure sucrose and fructose. The 24 h urinary sucrose and fructose biomarker (24hruSF) (mg/d) represents the sum of 24 h urinary sucrose and fructose excretion levels. The association between the 3-day mean total sugars intake and log 24uSF level was assessed using the Pearson correlation. A linear mixed model regressing log-biomarker on total sugars intake was used to investigate further the association between biomarker, diet, and other covariates. Mean (S.D.) total sugars intake for the group was 197.7 g/d (78.9). Log 24uSF biomarker was moderately correlated with total sugars intake (*r* = 0.33, *p* = 0.01). In stratified analyses, the correlation was strongest in females (*r* = 0.45, *p* = 0.028), the 18–30 age group (*r* = 0.44, *p* = 0.079), Indigenous Americans (*r* = 0.51, *p* = 0.0023), and the normal BMI category (*r* = 0.66, *p* = 0.027). The model adjusted for sex, age, body fat percent, and race/ethnicity demonstrated a statistically significant association between 24uSF and total sugars intake (β = 0.0027, *p* < 0.0001) and explained 31% of 24uSF variance (marginal R^2^ = 0.31). Our results demonstrated a significant relationship between total sugars intake and the 24uSF biomarker in this diverse population. However, the results were not as strong as those of controlled feeding studies that investigated this biomarker.

## 1. Introduction

Sugars, which include mono- and disaccharides, can lead to adverse health outcomes when eaten in excess [1,2]. Dietary sources of added sugars comprise mono- and disaccharides such as sucrose, fructose, maltose, and glucose. Sucrose and fructose are naturally found in foods like fruits and vegetables and are also added as common sweeteners to processed foods (e.g., high-fructose corn syrup) [3]. In the U.S., NHANES 2018 data reveal that Americans eat almost 17 teaspoons of added sugars daily, contributing roughly to 12.4% of intake for an average 2000 kcals diet [4]. This is above the WHO and Dietary Guidelines for Americans’ recommendation of <10% of daily kcal intake [5,6].

NHANES typically measures intake through the self-report of two non-consecutive 24 h recalls. Self-reported dietary intake is known to be associated with large measurement error [7]. Compared to doubly labeled water (DLW), a biomarker of energy intake, validity coefficients for self-report measures of energy intake from 24 h recalls and food frequency questionnaires (FFQ) are low (*r* = 0.23 and 0.24, respectively) [8]. Furthermore, using this biomarker, studies have shown strong and consistent underreporting of energy intake in self-reported measures in both adult and child populations (24 h recall = 10–28%; FFQ = 26–32%) [9]. Among energy-contributing sources, high-sugar foods are among the highest category of misreported food groups [10,11]. In a systematic review, Whitton et al. demonstrated that the omission rate of foods with added sugars was 40%, ranking among the highest food group categories in this analysis [11].

The inability to accurately assess dietary sugars impacts the quality of results in diet–disease association studies. In fact, a recent meta-analysis by Huang et al. mentioned that studies examining the relationship between dietary sugars intake and adverse health outcomes (e.g., cardiovascular disease, cancer, obesity) demonstrate “low” to “very low” quality evidence, especially concerning cancer [2]. Meta-analyses of sugars intake and adverse health outcomes like type 2 diabetes and cardiovascular disease have shown inconsistent results [12,13]. Both meta-analyses note some studies that show an increased risk of adverse health outcomes with high added sugars intake, while some demonstrate no association. While this inconsistency goes against conventional wisdom regarding the adverse effects of dietary sugars, it has been demonstrated that the measurement error in self-reported intake measures may be to blame for such discrepancies in the observed evidence [14].

Even though self-report measures of dietary intake are prone to bias, it is still important to collect this type of data from a large number of participants through epidemiological studies. Researchers can improve the quality of studies by developing biomarkers and applying them to assess measurement error in self-reported estimates, or correct disease risk estimates for measurement error, as previously demonstrated [15,16,17]. A biomarker for total sugars intake, known as 24 h urinary sucrose and fructose (24hruSF), has been developed [18]. In the GI tract, sucrose is first hydrolyzed to glucose and fructose, while fructose is readily absorbed into the gut as is and enters circulation [19]. However, trace amounts of fructose are filtered into the urine and excreted [19]. Researchers have identified a dose–response relationship between total sugars intake and urinary sucrose and fructose excretion levels [20,21].

This biomarker has previously demonstrated a significant association with diet in two highly controlled feeding studies [20,21], and has since been used in a number of population studies [16,22,23,24,25]. Although the biomarker has been previously investigated with a U.S. diet, the range of sugars intake was relatively narrow, and a majority of the population was predominantly White, with 17% Hispanics and 1% Indigenous Americans [21]. This study in a more diverse population offers an opportunity to investigate the biomarker over a wider range of sugars intake. Our aim is to test the performance of 24hruSF as a measure of total sugars intake in this diverse population during ad libitum dietary intake, using a vending machine paradigm. For this purpose, we used an inpatient feeding study conducted at the NIDDK Clinical Research Unit in Phoenix, Arizona, primarily designed to understand the influence of food preferences and intake on obesity in predominantly Indigenous American population [26].

## 2. Subjects and Methods

### 2.1. Study Recruitment and Data Collection

Sixty-four healthy adults were recruited at the NIDDK Clinical Research Unit in Phoenix, Arizona. Participants stayed in an inpatient facility during the ten-day duration of the primary study [26]. During participants’ inpatient stay (Flow Chart in Supplemental Figure S1), researchers collected pertinent data such as ad libitum dietary intake, 24 h urine, and body composition data through dual X-ray absorptiometry (DXA, DPX-1, Lunar Radiation Corp., Madison, WI, USA). Study staff also measured routine chemistry, such as serum creatinine, in the hospital laboratory (Dade Behring Dimension RxL Chemistry analyzer, Siemens Medical Solutions, Malvern, PA, USA). Collection methods for ad libitum dietary intake have been described below. Participants also spent approximately 24 h in a whole room calorimeter that collected energy expenditure and spontaneous physical activity (SPA), both of which have been previously discussed [27]. For the purposes of this study, we used data collected over the last three days of participants’ inpatient stay, including ad libitum dietary intake and 24 h urine samples, discussed in further detail below. The study received approval from the NIH Institutional Review Board (IRB), and all participants provided informed consent (ClinicalTrials.gov identifier: NCT00342732).

### 2.2. Ad libitum Dietary Intake Assessment

A vending machine paradigm measured ad libitum dietary intake over the last 3 days of the inpatient stay. This paradigm exhibits a high intraclass correlation coefficient (ICC = 0.90) and is thus reproducible [26]. To select foods that would be added to the vending machine, participants filled out the “Food Selection Questionnaire”, which contained a list of 77 food items. Examples of the range of food items include single items like hard-boiled eggs or oranges to complete, ready-to-eat meals like spaghetti and meatballs. A complete list of food items is included in the Supplement (see Supplemental Table S1). Participants rated these foods on a scale of 1–9 (1 being the lowest and 9 being the highest). Forty food items rated by participants between 4 and 8 were selected by research staff to fill a vending machine, one for each participant. This range was selected to ensure that participants received foods they liked while discouraging overeating by presenting them with foods they rated as highly desirable. Before food was added and after it was removed from each vending machine, research staff weighed and recorded all food items and wrappers/waste; thus, the entire dietary intake was known. This process was repeated every 24 h over the 3 days. The collected food intake data were entered into Food Processor (version 10.0.0; ESHA Research, Salem, OR, USA). This software program calculated the total caloric, fat, protein, carbohydrate, and total sugars intake for each participant.

### 2.3. 24 h Urine Collection and Urinary Sugars Biomarker Assessment

Participants collected 24 h urine samples for three consecutive days during the inpatient stay that overlapped with the vending machine protocol (days 7–9, Supplemental Figure S1). The 24 h urine collection started in the morning after the first void was discarded. Urine was collected for the next 24 h, beginning with the 2nd urine of the day and ending with the 1st urine of the following day. During the 24 h urine collection period, urine was kept cold in the fridge throughout the day.

Urine samples were sent to the NIDDK Clinical Laboratory Core for the measurement of sucrose and fructose using enzymatic assays (Enzytec Liquid Sucrose/D-Glucose and Enzytec Liquid D-Glucose/D-Fructose; R-Biopharm, Darmstadt, Germany). These assays were adapted to be run on a microplate reader, which included cutting the volumes by a factor of 10 and using 96-well plates. The limit of detection (LoD) for the adapted assays was 6.7 mg/L for fructose and 2.4 mg/L for sucrose. All urine samples that did not meet this sensitivity threshold were excluded from further analysis. Once a sample’s sugar concentration was determined, the biomarker could be calculated based on 24 h urine volume. To calculate the 24 h urinary sucrose and fructose biomarker (24hruSF), daily excretions of sucrose and fructose were summed up and expressed in mg/day.

### 2.4. Statistical Analysis

Statistical analyses were completed using SAS (version 9.4, SAS Institute Inc., Cary, NC, USA) and R (version 4.3.0, R Core Team, 2023, Vienna, Austria). As seen in Table 1, mean +/− standard deviation was used to express normally distributed continuous data, while absolute counts and percentages were used to express categorical data. Sex differences were assessed using independent samples *t*-tests. An alpha of 0.05 was set as the significance level for all tests. Distributions of all variables were checked for normality. Non-normal distributions were then log-transformed. The log-transformed variables for the biomarker and individual urinary sugars were chosen for the subsequent statistical analyses.

Initially, Pearson correlation coefficients (*r*) assessed the relationships between the 3-day mean of all variables, including the log 24hruSF (mg/d), urinary sucrose (mg/d), or urinary fructose (mg/d) vs. intake of total sugars, total carbohydrate intake, non-sugar carbohydrate (i.e., total carbohydrates minus total sugars), soda calorie intake, and body composition at baseline (by BMI and body fat %). Then, we stratified the correlations by relevant categorical variables, including sex (male and female), age category (18–30, 31–45, and >45), race/ethnicity (White, Indigenous Americans, Alaska Natives (AI/AN), and African American (AA)/other), and BMI category (Normal < 25, Overweight 25–29.9, and Obese ≥ 30).

As individuals had repeated measures across the 3 days, repeated measures correlation coefficients (*r*_m_) were also assessed using the Rmcorr package in R. Rmcorr accounts for the non-independence of repeated measures and can address within-subject correlations as opposed to the Pearson correlation coefficient described above, which assesses between-subject correlation. Rmcorr uses ANCOVA model varying intercepts and a common slope to yield a standardized coefficient that is interpreted identically to the Pearson r correlation coefficient.

Finally, the relationship between 24hruSF and sugars intake was examined using a linear mixed model with random subject intercept and assuming a first-order autoregressive covariance structure. The initial model included sex and age. Other covariates, such as race/ethnicity (AI/AN, White, AA, and Other (Hispanic, Asian, and people who identified as multiple races)), body fat %, physical activity, creatinine, protein intake, non-sugar carbohydrate intake, and fat intake were considered in the model. To investigate if multicollinearity in the fixed effects was an issue in the linear mixed model, we estimated the variance inflation factor (VIF) using the proc reg procedure in SAS. None of the variables tested in the backward selection models had a VIF > 4, indicating no collinearity. Hence, we proceeded with a backward selection model, which was applied to identify the final model by removing covariates that did not reach a significance of *p*-value > 0.05. The variance in the biomarker (24hruSF) explained by the model was calculated using the marginal R^2^ [28].

## 3. Results

The final analysis included 62 participants. Of the 512 urine samples, 31 were excluded as they did not meet the sensitivity threshold of the urinary sugars test. A majority of the participants were male (*n* = 37, 59.7%) and Indigenous American (*n* = 36, 58.1%) (Table 1). While both genders were similarly represented in AI/ANs and AAs, the majority of Whites were males. The average BMI of the study population was 30.59 (SD = 7.6), with most participants falling into either the overweight (41.3%) or obese (38.1%) BMI category. BMI categories, urine sugars, and biomarker concentration did not significantly differ by sex. Females had a higher body fat % (39.8 vs. 27.5, *p* < 0.0001) and fat mass (33.3 vs. 25.9, *p* = 0.042), while males had a higher fat-free mass (63.4 vs. 48.2, *p* < 0.0001) and were taller (174.1 vs. 159.2, *p* < 0.0001). Mean (S.D.) total sugars intake for the group was 197.7 g/d (78.9). Males had greater intake when compared to females in every intake category except for soda calories (*p* = 0.33).

**Figure 1:**
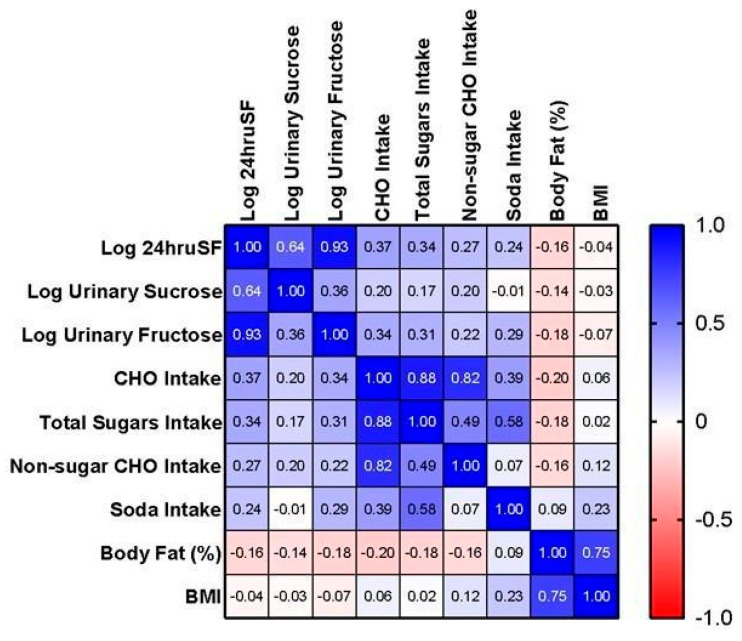
Pearson correlation coefficient (*r*) between biomarker and variables of interest. Heat maps demonstrating the correlations between biomarker (Log 24hruSF), dietary intake, and body composition measurements. All intake and urinary sugars variables represent the 3-day mean. Units for the variables are as follows: CHO intake = grams; total sugars intake = grams; non-sugar CHO intake = grams; soda intake = kcals; log urinary sucrose = mg/d; log urinary fructose = mg/d; and BMI = kg/m^2^.

Figure 1 shows Pearson correlation coefficient (*r*) between log biomarker and variables of interest. The 3-day means of log 24hruSF biomarker and total sugars intake were positively correlated (*r* = 0.34, *p* = 0.007). The correlation with total sugars was stronger for log urinary fructose (*r* = 0.31; *p* = 0.016) than for log urinary sucrose (*r* = 0.17; *p* = 0.20). The correlation between the log biomarker and other dietary variables were all significant: carbohydrate intake (*r* = 0.37, *p* = 0.003) and non-sugar carbohydrate intake (*r* = 0.27, *p* = 0.039) except soda intake (*r* = 0.24, *p* = 0.065). Interestingly, neither the biomarker nor total sugars were significantly related to either body fat % (*r* = −0.16; *p* = 0.21; *r* = −0.18; *p* = 164; respectively) or BMI (*r* = −0.04; *p* = 0.75; *r* = 0.02; *p* = 0.890; respectively).

Table 2 reports the correlation coefficients between the 3-day mean log 24hruSF and total sugars intake stratified by sex, race/ethnicity, age, and BMI. The strongest correlations were seen in females (*r* = 0.45, *p* < 0.05), AIs/ANs (*r* = 0.52, *p* < 0.01), and participants with normal BMI (*r* =0.66, *p* < 0.05). 

**Figure 2:**
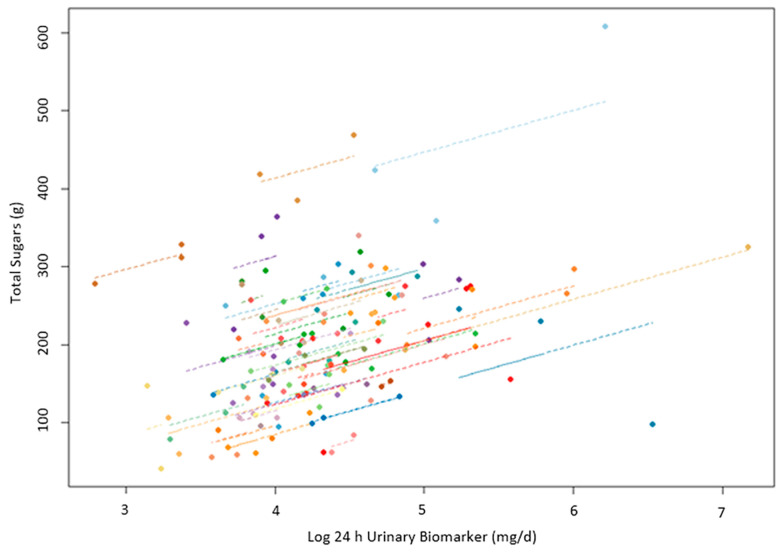
Within-subject repeated measures correlation. The correlation coefficient for repeated measures (*r_rm_*) comparing the biomarker (log 24hruSF) to total sugars intake within subjects (*r_rm_* = 0.44, *p*-value < 0.0001). Note that each participant is a different color dot representing 1–3 urine collections. Each line represents the correlation between daily 24hruSF biomarker and total sugars intake levels by individual (i.e., within-subject correlation).

The within-subject repeated measures correlations between the biomarker and total sugars intake, seen in Figure 2, were stronger (*r_rm_* = 0.44, *p* < 0.0001, 95% CI = 0.27–0.59) than the between-subject Pearson correlation coefficients shown, in Figure 1. The ICC (95% CI) for the biomarker was 0.60 (0.50–0.76) and 0.73 (0.63–0.82) for the total sugars intake.

As demonstrated in Table 3, total sugars intake was positively associated with the biomarker (β = 0.0027, *p* < 0.0001) after adjusting for race/ethnicity, age, sex, and body composition. AI/AN (ref = White, β = 0.57, *p* = 0.0017) and AA race/ethnicity (ref = White, β = 0.73, *p* = 0.0097) were also positive predictors of total sugars intake, while body fat % (β = −0.026, *p* = 0.015) was a negative predictor. This model, which included total sugars intake and covariates, explained 31% of the total variance in the biomarker (marginal R^2^ = 0.31). Twenty-one percent of this variance was explained by covariates, while an additional 10% by total sugars intake.

Other potential covariates, including SPA, protein intake, non-sugar carbohydrate intake, and fat intake, were removed from the model due to a lack of statistical significance. Additionally, to investigate whether kidney function affected the association between the biomarker and diet, serum creatinine was controlled for, and no effect was found on biomarker excretion. In sensitivity analyses, body fat % was replaced with BMI or fat mass and fat-free mass, which did not change any of the results.

## 4. Discussion

In this ad libitum dietary intake analysis, we demonstrated that the log 24hruSF biomarker was significantly related to total dietary sugars intake. Correlation coefficients demonstrated a significant positive relationship between dietary sugars intake and the biomarker. Stratified analyses by sex, race/ethnicity, and BMI revealed that the correlation between biomarker and total sugars intake was strongest in females, AI/AN, and normal weight participants. We also demonstrated that total sugars, race/ethnicity, and body composition were significant predictors of the biomarker.

Although significant, associations observed here were weaker than those reported by previous studies that tested the relationship between the biomarker and total sugars intake. According to Louie 2020 [3], an acceptable correlation coefficient for biomarkers in nutritional research is 0.5–0.7, which our biomarker did not reach. Previous feeding studies of a similar design investigating the 24hruSF biomarker as a predictor of dietary sugars intake have reported correlations over this threshold [20,21]. In another U.S. feeding study, the correlation coefficient between mean intake and biomarker was *r* = 0.68 [21] compared to 0.34 in our study. In an earlier feeding study conducted with a U.K. diet, the correlation coefficient between the biomarker and sugars intake was *r* = 0.84 [20]. Possible reasons for the lower correlations could be the fewer days of urinary and dietary data available in our study compared to those in prior feeding studies, where the collection of up to 30 days of urine samples and diet may have provided a better characterization of the biomarker and total sugars intake levels [20,21]. Increasing the number of 24hruSF biomarker repeats led to an improvement in the validity coefficient of the biomarker as a measure of sucrose plus fructose intake, and in a dietary validation study involving 198 free-living individuals conducted in the Netherlands that collected over period of three years two days of duplicate diets analyzed for sucrose and fructose and two 24 h urine samples analyzed for the 24hruSF biomarker [29]. The validity coefficient for the biomarker against the sum of sucrose and fructose intake ranged from 0.28 in women and 0.42 in men for the biomarker measured in one 24 h collection to 0.49 and 0.62, respectively, for infinite days of biomarker measurement [29]. No validity coefficient could be calculated for total sugars for comparison in our study, as no analytical values for total sugars were available from the duplicate diets. Even though results were weaker than those of previous studies assessing this biomarker, the biomarker still demonstrated a stronger association than self-report measures such as FFQ (*r* = 0.21–0.24), which is among the most widely used but most prone to error of the self-report measures [8].

The correlation between 24hruSF and total sugars intake in our study was similar to the correlation between the biomarker and non-sugar carbohydrate intake (0.34 vs. 0.27). In our study population, total sugars intake was positively correlated with non-sugar carbohydrate intake (*r* = 0.49), which may be the reason for the observed positive correlation between 24hruSF biomarker and non-sugar carbohydrate, despite the lack of biological plausibility for the association. Nonetheless, in the initial linear mixed model, non-sugar carbohydrate intake was included alongside other dietary and non-dietary covariates, and as expected, it was not found to be a significant predictor of the biomarker (estimate = 0.000917; *p* = 0.348).

In our study, the within-subject correlation, which is the average estimate for the correlation between diet and biomarker on the individual level, was somewhat stronger (*r* = 0.44) than the between-subject correlation (*r* = 0.34). In an earlier study, the within- and between-subject correlations were similar (0.69 and 0.68, respectively) [21]. While small, this within-person to between-person difference in correlation suggests some level of between-subject variability in the biomarker–diet association.

Our findings exhibit some similarities to earlier reports, albeit with a smaller effect size. Previously, a higher marginal R^2^ was reported in comparison to our model (0.56 vs. 0.31) even though both models incorporated similar covariates, including sex and age [21]. Notably, in both models, total sugars intake emerged as the biomarker’s strongest positive predictor. However, in a previous study, total sugars intake explained 52% of the 56% biomarker variance, but in our model, the explanatory power of total sugars was lower, at 10% of the total 31% [21].

However, unlike the previous analyses, our model did not find age, sex, or macronutrient intake to be significant positive predictors [21]. Instead, race/ethnicity (specifically AI/AN and AA) demonstrated significance as a positive predictor in our model. When stratified by race/ethnicity, the relationship between the biomarker and total sugars was only significant in the AI/AN group, not the White group. This is an interesting finding, given that several other feeding studies examining the biomarker demonstrated the biomarker’s effectiveness in a mostly White population [21] or all White populations [20,30]. Furthermore, in contrast to previous evidence, the correlation between 24hruSF and total sugars intake differed across BMI categories. In a randomized crossover feeding study, BMI was not found to have an effect on the biomarker–diet association across a wide range of total sugars intakes [30]. One potential reason for these inconsistencies with previous reports may be the lack of statistical power in our stratified analyses, given our study was not powered to investigate the performance of the biomarker across different subgroups.

Overall, total sugars intake in our population (197.7 g/d) was almost double than that reported in the previously mentioned U.S. feeding study (109.7 g/d) but was similar to the total sugars intake reported in the U.K. feeding study (202 g/d) [20,21]. Our study recorded a mean urinary fructose and sucrose excretion of 72.3 mg/d and 29.6 mg/d, respectively. In contrast, a U.K. feeding study reported lower values for urinary fructose (61.8 mg/d) and slightly higher values for urinary sucrose (36.6 mg/d) [20]. This difference can likely be attributed to the greater use of fructose as a sweetener in the U.S. compared to the U.K., where sucrose constitutes the primary type of sweetener consumed [3]. Thus, our higher fructose excretion and their slightly higher sucrose excretion can be explained by the dose–response relationship between intake and excretion of these types of sugars.

Determining a predictive biomarker for food and nutrition intake poses challenges, as individual differences in metabolism, digestion, and absorption can impact results [18]. Despite these limitations, predictive biomarkers like 24hruSF hold significance in epidemiology, particularly as they allow the generation of regression calibration equations that can alleviate the effect of measurement error and, consequently, improve the quality of research [31]. Additionally, previous reports have suggested that true associations between intake and biomarkers are more likely to be revealed in wider intake ranges [32]. However, despite our population’s wider range of sugars intake compared to previous reports (our SD = 78.9 vs. SD = 46.9), we found a weaker association between the biomarker and diet [21]. The other two feeding studies had more repeated measurements per participant (eight or more), which allowed for obtaining a better “true” estimate of one’s usual intake, while this study had only three [20,21]. Due to the nature of the study, a majority of the foods offered in the vending machine protocol were processed, branded, ready-to-eat foods for which the composition data originated from the Global Branded Food Products Database based on proprietary information provided by the industry [33]. These data are known to be less accurate than the other types of U.S. food composition data, e.g., the analytic nutrient values of the Foundation Foods generated in food composition laboratories [34], which may have further contributed to the low diet–biomarker correlation observed here.

A strength of our analysis was the sample size of over 60 participants, the diversity of the population with a large percentage of AI/AN population, and the objective ad libitum nature of the diet. By allowing participants to consume foods and beverages freely, this design may more closely reflect real-world eating behaviors. However, there were some limitations to this analysis as well. Although, in this study, we used an enzymatic spectrophotometric assay for biomarker measurement as in the previous feeding studies investigating this biomarker, the analytical approach used here had a lower sensitivity threshold due to assay modifications using much less sample volume. However, less than 10% of the samples at the low excretion range were excluded from the analysis. We also could not investigate the biomarker in relation to added sugars, fructose, or sucrose intake due to the high percentage of missing values (approximately 50%) of these nutrients for the vending machine foods/meals consumed by the participants in the Food Processor database used in this study. Lastly, we acknowledge that collecting 24 h urine is a burdensome process for both the participants and research staff and presents an important limitation of this biomarker. To address this problem, an approach using the biomarker measured in two-timed spot urine voids has been recently proposed [35].

There are several ways in which future studies could improve upon study design. In order to better characterize true usual intake and biomarker level, future studies could look at increasing the length of the objective vending machine period as well as days of urine collection. Lastly, a more balanced recruitment across sex, BMI, and ethnicity should be prioritized.

## 5. Conclusions

In conclusion, we demonstrated that the previously established 24 h urinary sucrose and fructose biomarker was positively associated with total sugars intake in this diverse population. However, the association in our population was weaker compared to that of previous reports. While this biomarker may still be better than the self-report measures of dietary intake, the reduced performance in this ad libitum intake paradigm requires further investigation.

## Figures and Tables

**Table 1:** Demographics and variables of interest.

Variable	Total	Female	Male	*p*-Value
Demographics, Body Composition, and other Covariates				
*n* (%)	62	25 (40.3)	37 (59.7)	
Age (years)	39.1 (12.5)	35.9 (12.1)	41.2 (12.5)	0.099
Age Category, *n* (%)				0.85
18–30	20 (32.3)	9 (36)	11 (29.7)	
31–45	20 (32.3)	8 (32)	12 (32.4)	
45+	22 (35.5)	8 (32)	14 (37.8)	
BMI (kg/m^2^)	30.6 (7.6)	32.2 (7.6)	29.5 (7.5)	0.18
BMI Category, *n* (%)				0.067
Normal weight	12 (20.6)	4 (16)	8 (21.6)	
Overweight	26 (41.3)	7 (28)	19 (51.4)	
Obese	24 (38.1)	14 (56)	10 (27)	
Race/ethnicity, *n* (%)				0.017 *
AI/AN	36 (58.1%)	19 (76%)	17 (45.9%)	
AA/Other	9 (14.5%)	4 (16%)	5 (13.5%)	
White	17 (27.4%)	2 (8%)	15 (40.5%)	
Body Fat (%)	32.5 (9.1)	39.8 (6.1)	27.5 (7.2)	<0.0001 **
Fat-free Mass (kg)	57.2 (11.6)	48.2 (7.9)	63.4 (9.5)	<0.0001 **
Fat Mass (kg)	28.9 (14.1)	33.3 (12.4)	25.9 (14.5)	0.0423 *
Height (cm)	168.0 (10.1)	159.2 (4.5)	174.1 (8.2)	<0.0001 **
Weight (kg)	86.1 (21.5)	81.5 (19.5)	89.3 (22.6)	0.16
Physical Activity (SPA)	7.9 (3.4)	7.5 (3.1)	8.2 (3.6)	0.51
Creatinine	0.8 (0.2)	0.7 (0.2)	0.9 (0.1)	<0.0001 **
Dietary Intake				
Total Sugars (g/d)	197.7 (78.9)	160.7 (53.9)	219.8 (83.8)	0.0028 **
Non-sugar CHO (g/d)	214.0 (65.9)	186.1 (59.4)	232.9 (64.1)	0.0052 **
Soda Intake (kcal/d)	198.5 (193.6)	169.2 (169.7)	218.3 (208.1)	0.33
Total CHO (g/d)	413.6 (126.7)	348.9 (91.0)	457.3 (129.6)	0.0006 **
Total Energy (kcal/d)	3141 (916)	2621 (693)	3492 (888)	<0.0001 **
Protein Intake (g/d)	99.3 (32.5)	77.1 (24.2)	114.2 (28.7)	<0.0001 **
Fat Intake (g/d)	123.8 (41.2)	104.4 (34.2)	136.8 (40.7)	0.0018 **
Urine Sugars				
Urinary Fructose (mg/d)	72.3 (80.6)	58.8 (31.0)	81.9 (101.8)	0.2774
Urinary Sucrose (mg/d)	29.6 (23.9)	22.3 (12.4)	34.5 (28.5)	0.0531
Biomarker				
24hruSF (mg/d)	101.7 (91.9)	79.2 (32.6)	117.5 (115.0)	0.1190

Values are expressed as means ± standard deviations unless specified otherwise. *p*-values demonstrate statistical significance between males and females, * *p* < 0.05; ** *p* < 0.01. BMI categories are <25 is Normal Weight, 25–29.9 is Overweight, and 30+ is Obese. All intake, urine sugars, and biomarker variables are reported as 3-day means. BMI= Body Mass Index. AA = African American. AI/AN = Indigenous Americans and Alaska Natives. Kcals = kilocalories. CHO = carbohydrate. Note that for physical activity (measured in SPA) there were 9 females with missing values and 10 males.

**Table 2:** Pearson correlation coefficient (*r*) between 3-day mean biomarker level and total sugars intake stratified by various demographics.

	N	*r* log 24hruSF vs. Total Sugars	*p*-Value
Sex			
Males	34	0.23	0.19
Females	24	0.45	0.028
Age category			
18–30	17	0.44	0.079
31–45	20	0.37	0.11
>45	21	0.31	0.17
Race/ethnicity			
White	16	0.21	0.43
AI/AN	33	0.52	0.0023
AA/other	9	0.21	0.59
BMI			
Normal	11	0.66	0.027
Overweight	23	0.073	0.74
Obese	24	0.53	0.0076

BMI categories are <25 is Normal Weight, 25–29.9 is Overweight, and 30+ is Obese. AA = African American. AI/AN = Indigenous Americans and Alaska Natives. Other includes Asians and those who identify as more than 1 race. Kcals = kilocalories. Units for the variables are as follows: total sugars intake = grams; age = years; and BMI = kg/m^2^.

**Table 3:** Regressing log 24hruSF biomarker on total sugars intake and other covariates in a linear mixed model.

Variable	Beta Estimate	*p*-Value	95% CI Lower	95% CI Upper
Total Sugars Intake (g/d)	0.0027	<0.0001	0.0016	0.0038
Race/ethnicity				
AI/AN	0.57	0.0017	0.22	0.91
AA	0.73	0.0097	0.18	1.28
Other	0.17	0.47	−0.30	0.65
Sex (Male)	0.0057	0.97	−0.35	0.36
Age	−0.0044	0.43	−0.015	0.0067
Body fat (%)	−0.026	0.015	−0.047	−0.0052

Race/ethnicity (ref = White). Sex (ref = female). AA = African American. AI/AN = Indigenous Americans and Alaska Natives. Other includes Asian and those who identify as more than 1 race. Units for the variables are as follows: 24hruSF = log; sugars intake = grams; and age = years.

## Data Availability

Due the enrollment of Indigenous Americans of Southwestern heritage in this study, data described in the manuscript, code book, and analytic code will be made available only upon request with approval from the principal investigator and via a tech transfer agreement with the requesting investigator and institution.

## Supplementary Materials

The following supporting information can be downloaded at: https://www.mdpi.com/article/10.3390/nu16050610/s1, Figure S1: Study Flow. Table S1: List of 77 Food Items Offered in the Vending Machine Protocol.
