# Supplementary material for: Twenty-Four-Hour Urinary Sugars Biomarker in a Vending Machine Intake Paradigm in a Diverse Population
Source: Nutrients. 2024 Feb 23;16(5):610. doi: 10.3390/nu16050610 (PMC10935035; doi:10.3390/nu16050610)
Supplement: Supplementary file 1 [file nutrients-16-00610-s001.zip › nutrients-2831522-supplementary.pdf]

Figure S1. Study Flow

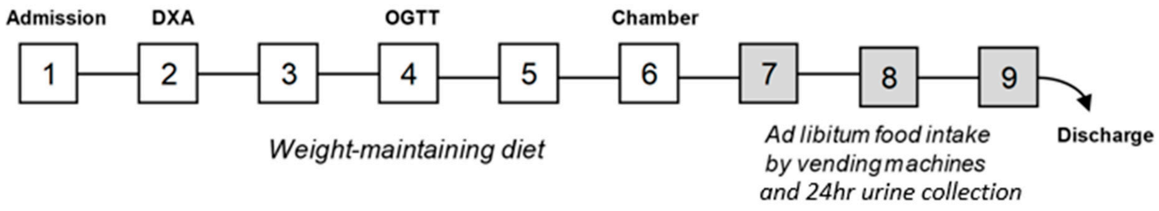

Table S1. List of 77 Food Items Offered in the Vending Machine Protocol		
**Food Item**		
Pizza	Oatmeal	Ham
Cheeseburger	Chocolate Pudding	Green Beans
Orange	English Muffin	Potato Salad
Fried Chicken	Corn Flakes and Milk	Chicken With Pasta
Eggs	Refried Beans	Popcorn
Baked Potato	Granola Bar	Apple Pie
Corn	Cup Of Noodles	Sliced Turkey
Spaghetti with Sauce	Bagel	Blueberry Muffins
Chicken Pot Pie	Rice Krispies and Milk	KitKat Bar
Barbecue Wings	Pinto Beans	Nestle Crunch
French Toast	Peanuts	Cheez-Its
Beef Stew	Sausage McMuffin	Tortilla Chips
Sausage	Ritz Crackers	Chicken Noodle Soup
Reese’s Cups	Rice Krispies Treats	Crackers
Cheddar Cheese	Cinnamon Bun	Pretzels
Chili With Beans	Cottage Cheese	Baby Ruth
Pancakes	Chocolate Donut	Spinach
Apples	Jello	Pork and Beans
Chicken Nuggets	Apple Sauce	Bologna
Stuffed Baked Potato	Croissant	Raisins
Potato Chips	Yogurt	Fig Newtons
Peanut M and M’s	Corned Beef Hash	Gummy Bears
Cooked Rice	Cheesecake	Fruit Roll-Ups
Tater Tots	Macaroni Salad	Mushroom Soup
Peaches	Canned Tuna	Sugar Wafers
Doritos	Fudge Cookies	
